# Dietary Red and Grey Selenium Nanoparticles: Effects on Tissue Selenium Distribution, Antioxidant Capacity, and Retention in Japanese Quails

**DOI:** 10.3390/antiox15010004

**Published:** 2025-12-19

**Authors:** Aya Ferroudj, Arjun Muthu, Georgina Pesti-Asbóth, Daniella Sári, Gréta Törős, Áron Beni, Levente Czeglédi, Renata Knop, Hassan El-Ramady, József Prokisch

**Affiliations:** 1 Nanofood Laboratory, Department of Animal Husbandry, Faculty of Agricultural and Food Sciences and Environmental Management, Institute of Animal Science, Biotechnology and Nature Conservation, University of Debrecen, 138 Böszörményi Street, 4032 Debrecen, Hungary; arjun.muthu@agr.unideb.hu (A.M.); saridaniella91@gmail.com (D.S.); toros.greta@agr.unideb.hu (G.T.); 2 Doctoral School of Animal Husbandry, University of Debrecen, Böszörményi Street 138, 4032 Debrecen, Hungary; 3 Institute of Agricultural Chemistry and Soil Science, Faculty of Agricultural and Food Sciences and Environmental Management, University of Debrecen, 138 Böszörményi Street, 4032 Debrecen, Hungary; beniaron@agr.unideb.hu; 4 Center for Complex Systems and Microbiome Innovations, Faculty of Agricultural and Food Sciences and Environmental Management, University of Debrecen, 4032 Debrecen, Hungary; georgina.asboth@agr.unideb.hu; 5 Department of Animal Husbandry, Faculty of Agriculture and Food Sciences and Environmental Management, University of Debrecen, 138 Böszörményi Street, 4032 Debrecen, Hungary; czegledi@agr.unideb.hu (L.C.); dr.knop.renata@agr.unideb.hu (R.K.); 6 Soil and Water Department, Faculty of Agriculture, Kafrelsheikh University, Kafr El-Sheikh 33516, Egypt; hassan.elramady@agr.kfs.edu.eg

**Keywords:** SeNPs, amorphous, crystalline, GPx, SOD, TAC, bioavailability, excretion, storage

## Abstract

This study evaluated the bioavailability, antioxidant response, and post-withdrawal retention of red and grey selenium nanoparticles (SeNPs) in adult male Japanese quails. Birds were fed a basal diet supplemented with 0.5 or 5 mg/kg of red or grey SeNPs for 28 days, followed by a 7-day withdrawal period. Selenium distribution varies markedly by nanoparticle form and dose. Red SeNPs, particularly at 5 mg/kg, produced higher selenium accumulation in metabolic and circulating tissues, whereas grey SeNPs showed lower initial uptake but more selective deposition at specific sites. Antioxidant analysis revealed significant increases in hepatic GPx activity across all SeNP groups, with the strongest enhancement occurring at the 5 mg/kg level. Serum TAC was elevated predominantly in quails receiving high-dose red SeNPs. Retention–depletion analysis demonstrated that moderate doses supported stable selenium incorporation, whereas high doses resulted in accelerated post-withdrawal loss. Overall, red SeNPs acted as rapidly available selenium sources with pronounced antioxidant effects, while grey SeNPs provided slower, more sustained selenium delivery. These findings highlight the importance of nanoparticle form and dosage in optimizing selenium supplementation strategies for poultry.

## 1. Introduction

Selenium (Se) is an essential trace element required for the synthesis of selenoproteins involved in antioxidant defense, immune function, and reproductive performance in birds [1]. In poultry nutrition, selenium supplementation is routinely used to strengthen oxidative stability, support metabolic health, growth performance, and egg production [2,3,4]. However, the bioavailability and safety of traditional inorganic and organic selenium sources vary considerably, motivating the search for more efficient and less toxic alternatives [5,6,7]. Selenium nanoparticles (SeNPs) have emerged as a promising form of supplemental selenium due to their lower toxicity, high surface reactivity, and ability to enhance antioxidant capacity at relatively low doses [8,9,10]. Recent studies across different stages of poultry production have demonstrated that nano-selenium supplementation can improve feed conversion efficiency, daily feed intake, average daily gain, carcass yield, and immune responsiveness, including enhanced resistance to bacterial challenges such as Salmonella Typhimurium [11,12]. These findings highlight the growing interest in nano-selenium as a functional nutritional strategy for improving poultry health and productivity. Among nano-selenium forms, red and grey SeNPs represent two distinct allotropes with fundamentally different physicochemical and biological properties [13,14,15]. Red SeNPs are typically amorphous or crystalline, forming spherical or rod-like particles, which are produced mainly through reduction of selenite by agents such as ascorbic acid or hydrosulfite, microwave irradiation, or chemical methods. Their amorphous structure confers high solubility, rapid dissolution, and increased surface reactivity, which contribute to their higher immediate bioavailability and functional incorporation into metabolic pathways [16,17,18,19,20,21,22]. In contrast, grey SeNPs correspond to the thermodynamically stable hexagonal crystalline phase composed of polymeric selenium chains and six-membered rings, typically formed upon heating, vaporization, sublimation, or high-pressure conversion of the red allotrope [23,24,25,26,27]. Grey SeNPs exhibit greater structural order, lower solubility, and slower release kinetics, functioning more as a sustained selenium reservoir than a rapidly absorbed source [28,29,30,31]. Despite extensive characterization efforts, nanoscale mechanisms governing the red-to-grey transformation and its biological implications remain incompletely understood [32,33,34,35,36,37,38]. These differences suggest that nanoparticle form may influence absorption, tissue distribution, and overall selenium utilization, yet comparative information in poultry remains limited. Understanding the metabolic fate of different SeNP forms is essential for evaluating their nutritional and physiological value. Tissue selenium accumulation patterns provide direct insight into bioavailability, while antioxidant biomarkers such as glutathione peroxidase (GPx), superoxide dismutase (SOD), and total antioxidant capacity (TAC) reflect their functional incorporation into biological systems [1,2,39]. Moreover, monitoring selenium retention and depletion following dietary withdrawal can reveal whether selenium is rapidly mobilized or retained as a slow-release reserve. This study aimed to compare the effects of red and grey selenium nanoparticles, provided at two dietary concentrations, on selenium distribution, antioxidant responses, and post-withdrawal retention in adult male Japanese quails. By integrating tissue deposition patterns with enzymatic and non-enzymatic antioxidant markers, our work provides new insight into how nanoparticle form and dose shape selenium bioavailability and physiological efficacy in poultry.

## 2. Materials and Methods

The study was conducted at the Nanofood laboratory of the Institute of Animal Science, Biotechnology and Nature Conservation, Department of Animal Husbandry, Faculty of Agricultural and Food Sciences and Environmental Management, University of Debrecen, Hungary. It was approved by the institutional ethics committee of the University of Debrecen (ethical permission number: 4/2021/DEMÁB). All methods were performed following the relevant guidelines and regulations.

### 2.1. Reagents

Sodium selenite, Vitamin C, nitric acid 65% (AR grade), hydrogen peroxide, and hydrochloric acid 37% (AR grade) were obtained from VWR, International Ltd. (Lutterworth, Leicestershire, UK). Sodium borohydride 98% (AR grade) was purchased from Acros Organics (Geel, Belgium).

### 2.2. Selenium Nanoparticle Preparation and Characterization

Red and grey selenium nanoparticles (SeNPs) were prepared according to the protocol described in [9]. Red SeNPs were synthesized by reducing sodium selenite (Na_2_SeO_3_) with 1% ascorbic acid at room temperature for 30 min, producing a stable red colloidal solution. Grey SeNPs were generated by thermally converting the red particles at 85~95 °C for 2 h, inducing their transition into the hexagonal crystalline form. According to the characterization report in [9], red SeNPs measure approximately 80–120 nm, whereas grey SeNPs range from 90–150 nm according to TEM and DLS analyses. The UV–Vis spectra should show a purity exceeding 95%, with no detectable residual inorganic selenium species. All physicochemical properties were reproduced and confirmed in our laboratory using the same synthesis procedure.

### 2.3. Experimental Design

A total of 60 adult male Japanese quails (Coturnix japonica; 12 weeks of age) were used in 28-day and 35-day feeding trials. Birds were individually housed in wire cages under standardized environmental conditions (temperature: 25 ± 2 °C; photoperiod: 16 h light/8 h dark) to allow for precise monitoring of feed intake and health status. All birds had free access to feed and water throughout the experimental period, with approximately 18 g of feed offered per bird per day. Quails were randomly allocated into five dietary treatment groups (n = 12 per group) based on initial body weight to ensure uniformity among groups. The treatments were as follows:

C0 (Control): Basal diet without selenium nanoparticle (SeNP) supplementation.

T1: Basal diet supplemented with 0.5 mg/kg red SeNPs.

T2: Basal diet supplemented with 5 mg/kg red SeNPs.

T3: Basal diet supplemented with 0.5 mg/kg grey SeNPs.

T4: Basal diet supplemented with 5 mg/kg grey SeNPs.

The basal diet (Table 1) was formulated using soybean, corn, wheat and sunflower oil considering the nutrient requirements of breeder quails according to [40]. The premix included in the basal diet provided a background selenium content of 0.042 mg/kg. Consequently, the total selenium content in the diets was estimated to be 0.042 mg/kg (C0), 0.542 mg/kg (T1 and T3), and 5.042 mg/kg (T2 and T4). After 28 days of dietary supplementation, half of the birds from each treatment group (n = 6) were slaughtered for sample collection. The remaining birds continued for an additional 7 days on the unsupplemented basal diet (Nano selenium-free phase) and were slaughtered on day 35. This design allowed for the evaluation of both immediate and residual effects of dietary SeNP supplementation following a withdrawal period. Feed intake was recorded daily for each cage throughout the experimental period and expressed as g/bird/day.

### 2.4. Tissue Sampling

The birds (n = 6 per treatment group) were randomly selected and euthanized after 28 days. Tissue samples were taken from the following organs (n = 6): liver, kidney, blood (which was centrifuged in anticoagulant tubes at 3000 rpm for 15 min to separate red blood fraction (cells) and serum), testis, spleen, breast and eyes, which were washed using phosphate-buffered saline solution (PBS) and stored at −80 °C until analyzed. The samples (0.5 g) were digested with 2.25 mL of concentrated HNO_3_ (65%) and 6.75 mL of concentrated HCl (37%), and then heated for 4 h at 80 °C. Selenium measurements were conducted using a Millennium Excalibur 10.055 atomic fluorescence spectrophotometer (AFS) (PSA, Orpington, UK).

The total retention % and depletion % of Selenium are the combined Se remaining in these organs (liver, kidney, red blood fraction (RBF), spleen) at 35 days from the remaining 30 birds, which were compared with the values from the same organs after 28 days according to the following formulas:

$$Total\;retention\;\%\;=\left(\frac{\sum \left(\mathrm{Se}\;\mathrm{in}\;\mathrm{organ}\;\mathrm{after}\;\mathrm{withdrawal}\;(35d)\right)}{\sum \left(Se\;in\;organ\;before\;withdrawal\;(28d)\right)}\right)\times 100$$

$$Se\;depletion\;\%=\left(1-\frac{\sum \left(\mathrm{Se}\;\mathrm{in}\;\mathrm{organ}\;\mathrm{after}\;\mathrm{withdrawal}\right)}{\sum \left(Se\;in\;organ\;before\;withdrawal\right)}\right)\times 100$$

### 2.5. Antioxidant Biomarkers

Antioxidant indices were determined by measuring the levels of glutathione peroxidase (GSH-Px), superoxide dismutase (SOD), and total antioxidant capacity (T-AOC). Liver homogenates were used to measure GSH-Px activity, while both liver and serum samples were analyzed for SOD and T-AOC. The following commercial assay kits were used: Invitrogen™ Glutathione Peroxidase (GSH-Px) Activity Kit (Cat. No. EEA010, Thermo Fisher Scientific, Waltham, MA, USA), Invitrogen™ Superoxide Dismutase (SOD) Colorimetric Activity Kit (Cat. No. EIASODC, Thermo Fisher Scientific, USA), and Antioxidant Assay Kit (Cat. No. KA1622, Abnova, Taipei, Taiwan). Absorbance was measured using a SPECTROstar Nano Microplate Reader (BMG LABTECH GmbH, Ortenberg, Germany).

### 2.6. Statistical Analyses

All statistical analyses were performed using GraphPad Prism version 9.5.0. Data are presented as the mean ± standard error of the mean (SEM). Selenium concentrations in tissues were analyzed using two-way analysis of variance (ANOVA) with selenium nanoparticle form (red vs. grey) and dietary dose (0, 0.5, and 5 mg/kg) as fixed factors. Analyses were conducted separately for each organ. Feed intake, antioxidant parameters (GPx, SOD, and total antioxidant capacity), and total selenium content were analyzed using one-way ANOVA with dietary treatment as the independent factor. Selenium distribution among organs within a single treatment was analyzed using one-way ANOVA with organ as the factor. When significant effects were detected (*p* < 0.05), Tukey’s multiple comparison test (HSD) was applied.

## 3. Results

The effects of dietary selenium nanoparticles on selenium distribution, feed intake, antioxidant activity, and retention dynamics were evaluated in adult male Japanese quails. The results are organized to first describe tissue selenium deposition across treatments, followed by changes in antioxidant biomarkers, and finally the patterns of selenium retention and depletion after the withdrawal period.

Table 2 shows the selenium distribution in organs of Japanese quails and total selenium content after 28 days of SeNP supplementation. Selenium concentrations varied significantly among treatments and organs (*p* < 0.0001). The red SeNP treatments (T1 and T2) resulted in higher selenium concentrations in several metabolically active tissues, with the highest values observed in the T2 group. In this group, selenium levels reached 263.18 µg/kg ± 26 in red blood fractions, 128.86 µg/kg ± 1.6 in the liver, and 196.93 µg/kg ± 6.2 in breast muscle, indicating a dose-dependent increase for red SeNPs. The lower red SeNP dose (T1) also increased selenium concentrations in the spleen, RBFs and breast compared with the control. Grey SeNP supplementation showed a distinct accumulation pattern. The low dose (T3) resulted in lower selenium concentrations in the spleen and testis while maintaining comparable breast muscle selenium levels to the red SeNP groups. At the higher dose (T4), grey SeNPs produced marked increases specifically in the spleen and testis, whereas liver selenium levels were comparable to those observed in T2. Kidney selenium concentrations varied moderately among treatments, with a significant elevation only observed in the T2 group relative to the control. Selenium concentrations in the eyes remained relatively stable across all treatments. Moreover, the high-dose groups T2 and T4 showed higher total Se levels compared with the control. Overall, the groups supplemented with nanoparticles of selenium produced the highest selenium accumulation and distribution in different organs; grey SeNPs demonstrated a strong dose dependence, with substantial increases observed only at higher concentrations in the spleen and testis.

**Figure 1:**
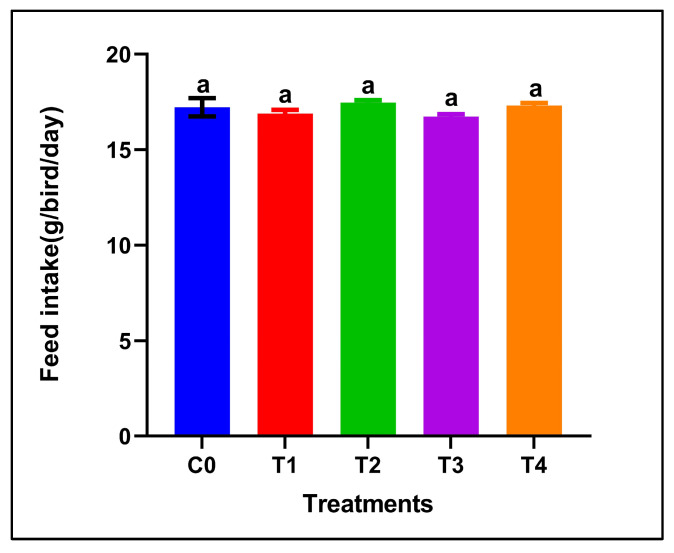
Feed intake of adult male Japanese quails during 28 days of dietary supplementation with selenium nanoparticles. Values are presented as mean ± SEM. No significant differences were observed among treatments (*p* > 0.05).

Dietary supplementation with red or grey SeNPs did not significantly affect feed intake throughout the 28-day feeding period (*p* > 0.05) (Figure 1). The mean FI remained comparable between the control and all supplemented groups, including the high-dose treatments (5 mg/kg), indicating normal feeding behavior and the absence of overt selenium-induced anorexia.

**Figure 2:**
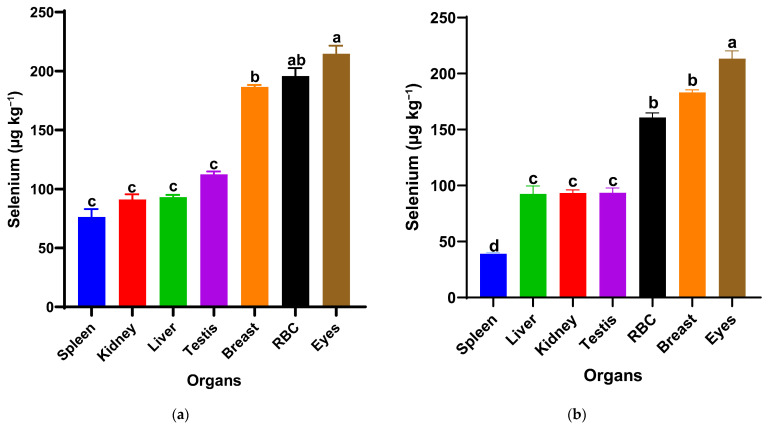
Selenium distribution in spleen, kidney, liver, testis, breast muscle, red blood cells (RBCs), and eyes of Japanese quails supplemented with red SeNPs ((**a**): T1, 0.5 mg/kg) and grey SeNPs ((**b**): T3, 0.5 mg/kg). Bars represent mean ± SEM. Different superscript letters within each treatment indicate significant differences in selenium concentration among organs (*p* < 0.05).

The selenium distribution notably differed between the two nanoparticle forms at the same dietary dose (0.5 mg/kg) in Figure 2. In the red SeNP group (T1) Figure 1a, selenium concentrations varied significantly among organs (*p* < 0.05). Red blood fraction selenium concentrations did not differ significantly from those in the eyes or breast muscle, whereas a significant difference was observed between the eyes and breast muscle, with the overall pattern being eyes ≥ RBF ≥ breast muscle > testis = liver = kidney = spleen. The highest levels were observed in the eyes, followed by the red blood fraction (RBF) and breast muscle, indicating relatively strong systemic availability and consistent deposition across tissues. The remaining visceral organs and testis showed lower but similar selenium concentrations (93–112 µg/kg), suggesting a homogeneous distribution pattern at this dose. In contrast, birds receiving the same dose of grey SeNPs (T3) in Figure 1b displayed a distinct deposition hierarchy of eyes > breast muscle = RBF > liver = kidney = testis > spleen (*p* < 0.05). While selenium levels in the eyes and breast muscle were comparable to those in T1, RBF selenium was notably lower, and spleen selenium accumulation was the lowest among all measured tissues (39.10 µg/kg ± 1). Overall, red SeNPs (T1) produced a slightly higher and more evenly distributed selenium profile across organs, whereas grey SeNPs (T3) resulted in reduced accumulation in circulating and immune tissues despite maintaining similar levels in muscle and ocular tissues.

**Figure 3:**
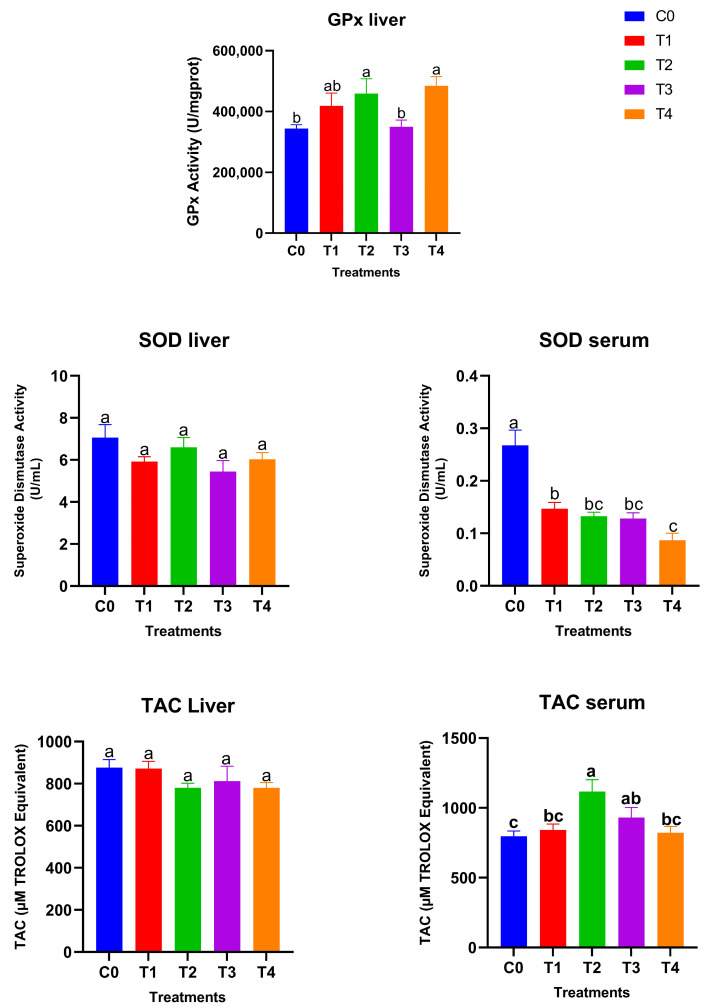
Antioxidant biomarkers in liver and serum of Japanese quails fed diets supplemented with selenium nanoparticles (SeNPs). Data are presented as mean ± SEM. Different superscript letters indicate statistically significant differences among dietary treatments (*p* < 0.05).

Figure 3 shows the glutathione peroxidase (GPx) activity, superoxide dismutase (SOD) levels, and total antioxidant capacity (TAC) in liver and serum samples after 28 days of treatment. Hepatic GPx activity responded strongly to dietary selenium supplementation. The high-dose SeNP-treated groups showed significantly higher GPx activity compared with the control and low grey Se treatment T3, while T1 showed an intermediate value (*p* < 0.05), with the greatest increases observed in T2 (red SeNP, 5 ppm) and T4 (grey SeNP, 5 ppm). These findings indicate a clear dose-dependent enhancement of GPx-mediated antioxidant capacity in the liver, irrespective of nanoparticle form. In contrast, hepatic SOD activity remained statistically unchanged among treatments, suggesting that liver superoxide dismutase activity is relatively stable and not markedly influenced by selenium level or nanoparticle type. Serum SOD activity exhibited an opposite trend: all SeNP-supplemented birds showed significantly lower serum SOD activity than the control group (*p* < 0.05); the activity levels were still maintained within the normal reference range, suggesting that the improvement in selenium status reduced the systemic demand for circulating SOD. Liver TAC (Total antioxidant activity) values did not differ significantly among treatments, indicating that hepatic non-enzymatic antioxidant capacity was maintained across dietary groups. However, serum total antioxidant capacity differed significantly among treatments (*p* < 0.05). The highest serum TAC was observed in T2, which was significantly higher than the control, T1, and T4, but did not differ significantly from T3. In addition, the T3 group exhibited significantly higher serum TAC compared with the control, whereas no significant differences were detected among the control, T1, and T4 groups. Taking together, these results demonstrate that selenium nanoparticles enhanced antioxidant status primarily through GPx upregulation and improved systemic TAC, with red SeNPs at 5 ppm exerting the strongest overall effect. Grey SeNPs also increased GPx activity, but their impact on serum antioxidant capacity was less pronounced with high supplementation, indicating comparatively lower bioefficacy at equivalent high doses.

Table 3 shows the total retention and depletion rate (in liver, kidney, spleen and red blood fraction RBF). The total selenium retention and depletion percentages revealed clear treatment-dependent patterns. Control birds exhibited the most stable selenium balance (96% retention, 3.9% depletion), reflecting normal homeostatic regulation. Among the supplemented groups, the low-dose treatments (T1 and T3) achieved the highest overall retention (91% and 88%, respectively), indicating efficient incorporation and stable maintenance of selenium during the withdrawal period. In contrast, higher doses (T2 and T4) showed markedly lower retention (78% and 57%). This drop is a direct consequence of the body reaching tissue saturation, which subsequently triggers enhanced excretion and biological regulatory mechanisms to rapidly clear the excess selenium once supplementation is withdrawn. Red SeNPs displayed higher initial bioavailability and retention overall (T1 was 91%), while grey SeNPs followed closely in second place, confirming that both nanoparticle forms lead to efficient selenium incorporation, but the clearance rate was primarily governed by the dose.

Although red SeNPs exhibited the highest selenium retention and antioxidant enhancement, the grey form also demonstrated notable biological activity, indicating that selenium absorption and utilization persist even after the red-to-grey transformation. This transformation reflects structural stabilization rather than degradation; therefore, the conversion does not mark the end of the red SeNPs’ shelf life. Instead, the newly formed grey crystalline particles continue to act as sustained selenium reservoirs, gradually releasing selenium within tissues. The maintained, though reduced, selenium accumulation and antioxidant response in grey SeNP-treated groups suggest that both nanoparticle forms are metabolically active—red SeNPs provide rapid and high bioavailability, while grey SeNPs ensure longer-term selenium provision and stability.

## 4. Discussion

This study demonstrated that selenium nanoparticles exerted clear form- and dose-dependent effects on selenium deposition, post-withdrawal retention, and antioxidant responses in adult male Japanese quails [41,42,43,44]. Across all evaluated parameters, red and grey SeNPs behaved differently, reflecting their contrasting dissolution rates, bioavailability, and tissue affinities [12,45,46,47]. Red SeNPs, particularly at 5 mg/kg (T2), consistently produced the highest selenium levels in metabolic and circulating compartments, including the liver, red blood fraction (RBF), and breast muscle [17,48,49,50]. This pattern aligns with the known higher reactivity and faster dissolution of amorphous red selenium, enabling efficient intestinal absorption and rapid incorporation into selenoproteins [51]. The pronounced increase in hepatic GPx activity and elevated serum TAC observed in T2 further supports the notion that red SeNPs provide readily available selenium for antioxidant enzyme synthesis and systemic redox balance [52]. Despite the supranutritional selenium level used in the 5 mg/kg treatments, no clinical signs of selenium toxicity were observed during the 28-day feeding period as the birds maintained normal feed intake, normal behavior, and good overall health status. However, the low-dose Red SeNPs (T1) demonstrated high initial bioavailability and also the highest overall retention (91%) among all supplemented groups following dietary withdrawal, indicating highly efficient incorporation and maintenance [53]. In contrast, grey SeNPs exhibited slower and more selective accumulation patterns [14,50]. At the low dose (T3), grey SeNPs resulted in generally lower organ selenium concentrations compared with the equivalent red SeNP dose, confirming reduced bioavailability at small doses [50]. Nonetheless, T3 birds retained selenium more consistently during the withdrawal phase than T2 and T4, suggesting slower release and more prolonged tissue retention, but low-dose Red SeNP-treated T1 birds showed superior retention compared to T3 and showed the most stable maintenance overall during the withdrawal phase [54]. At the high dose (T4), grey SeNPs produced strong accumulation in specific organs, most notably in the spleen and testis, while showing a marked decrease in total retention after withdrawal, likely reflecting saturation of the storage capacity followed by compensatory mobilization or excretion of excess selenium after supplementation ceased. These findings indicate that higher selenium doses do not necessarily improve long-term retention and may reduce supplementation efficiency, highlighting the importance of moderate dosing to achieve sustained selenium status while maintaining safety [55,56]. Across all treatments, the eyes consistently displayed the highest selenium concentrations, independent of SeNP form or dose. This stability suggests a strong physiological requirement for selenium in ocular antioxidant defenses, likely driven by high GPx expression and the sensitivity of retinal tissues to oxidative stress [57,58]. Meanwhile, the spleen and visceral organs showed greater variability among treatments, indicating dynamic selenium redistribution associated with immune and metabolic processes [59,60]. The retention–depletion analysis provided further insight into the kinetic differences between nanoparticle types. Low doses (T1 and T3) resulted in the highest overall retention, whereas high doses (T2 and T4) were associated with accelerated clearance following the withdrawal period [55,57,61]. This dose-dependent clearance pattern suggests that moderate selenium supplementation supports stable tissue incorporation, while higher doses trigger homeostatic regulation and enhanced excretory responses to maintain physiological selenium balance [55,56,61]. Red SeNPs supported greater overall bioavailability and maintenance, with the low dose (T1) achieving the highest total retention among all supplemented groups. Grey SeNPs—particularly at the low dose—provided a sustained release profile that ensures longer-term availability due to their structure. Collectively, these findings highlight that selenium form and dose strongly influence the balance between absorption, utilization, tissue storage, and clearance in quails. Red SeNPs excelled in rapid antioxidant enhancement and systemic distribution, and had superior overall retention. In contrast, grey SeNPs showed distinct advantages in slower, targeted accumulation (spleen/testis) and a release mechanism designed for prolonged selenium provision [62,63].

## 5. Limitations and Perspectives

While the present study provides a robust physiological evaluation of red and grey selenium nanoparticles in adult male Japanese quails, several factors warrant further investigation. The responses observed here may vary with age, sex, and species, as selenium metabolism and antioxidant requirements are influenced by developmental stage, reproductive status, and genetic background. Additionally, environmental conditions, such as temperature, housing density, and oxidative stress load, may modulate selenium utilization and retention. Although growth performance has been addressed in our previous low-dose studies [41], the supranutritional level (5 mg/kg) examined here requires extended evaluation to define long-term safety margins. Future research should integrate comprehensive physicochemical characterization of nanoparticles alongside molecular analyses, including selenoprotein- and antioxidant-related gene expression, to clarify the underlying mechanisms. Moreover, exploring combined supplementation strategies, such as selenium nanoparticles with vitamin E or other bioactive compounds at varying doses, may provide synergistic benefits and improve nutritional provision. Collectively, these approaches will support the rational optimization of selenium nanoparticle use in poultry nutrition.

## 6. Conclusions

Selenium nanoparticles influenced selenium distribution and antioxidant status in quail tissues in a form- and dose-dependent manner. Red SeNPs demonstrated higher overall bioavailability, resulting in superior selenium deposition in metabolic tissues and stronger systemic antioxidant support (e.g., increased serum Total Antioxidant Capacity). In contrast, grey SeNPs exhibited a slower release profile and more selective accumulation at specific sites (e.g., spleen and testis), suggesting an advantage for prolonged selenium delivery. All SeNP treatments successfully enhanced the primary enzymatic defense (GPx), though moderate doses produced the most stable tissue incorporation, while higher doses accelerated selenium clearance due to tissue saturation. Ultimately, the study highlights the distinct and complementary properties of the red and grey SeNP forms, underscoring the necessity of selecting both the nanoparticle form and dose to achieve targeted nutritional and antioxidant benefits for poultry.

## Figures and Tables

**Table 1:** Ingredients and nutrient composition of the diet.

Feed Ingredient	Inclusion Rate, %
Soybean meal (46% CP)	34.88
Corn	30.37
Wheat	20.00
Sunflower oil	6.79
Limestone	5.64
MCP	1.29
Salt	0.38
DL-Methionine	0.15
Vitamin and mineral premix ^a^	0.50
Nutrient content, %	
Metabolisable energy (MJ/kg)	12.13
Crude protein	20.0
Calcium	2.50
Available Phosphorus	0.35
Sodium	0.15
Methionine	0.45
Methionine + cysteine	0.75
Lysine	1.08
Threonine	0.74
Leucine	1.59
Isoleucine	0.86
Arginine	1.33
Tryptophan	0.25

^a^ 1 kg of premix provides 1,000,000 NE vitamin A, 200,000 NE vitamin D3, 4900 mg/kg vitamin E, 200 mg vitamin K3, 150 mg vitamin B1, 500 mg vitamin B2, 1200 mg Ca-d-Pantothetane, 400 mg vitamin B6, 2 mg vitamin B12, 11 mg biotin, 2502 mg niacin, 60 mg folic acid, 300,000 mg choline chloride, 13,200 mg Zn, 1920 mg Cu, 9612 mg Fe, 13,200 mg Mn, 180 mg I, 42 mg Se, 12 mg Co.

**Table 2:** Selenium distribution in organs of Japanese quails and total selenium content after 28 days of SeNP supplementation.

Se Content (μg.kg^−1^)	C	SEM	T1	SEM	T2	SEM	T3	SEM	T4	SEM	*p* Value
**Spleen**	49.3 ^c^	1.6	76.3 ^b^	6.6	67.7 ^b^	4.6	39.1^d^	1.02	135.2 ^a^	3.8	<0.0001
**Kidney**	101.3 ^b^	3.7	91.2 ^b^	4.4	114.8 ^a^	1.5	93.4 ^b^	2.8	89.6 ^b^	4.4	<0.0001
**Testis**	119 ^b^	5.3	112.4 ^b^	2.5	124.2 ^b^	3.1	93.5 ^c^	4.3	151 ^a^	7.2	<0.0001
**Eyes**	220.4 ^a^	7.1	214.7 ^a^	6.9	240.8 ^a^	7.7	213.4 ^a^	6.9	222.1 ^a^	4.5	<0.0001
**RBF (red blood fraction)**	166.5 ^c^	4.9	195.9 ^b^	6.7	263.2 ^a^	27	160.8 ^c^	4	162.4 ^c^	3.8	<0.0001
**Breast muscle**	178.8 ^b^	1.5	186.6 ^ab^	1.6	196.9 ^a^	6.2	183.2 ^ab^	2.3	192.1 ^ab^	9.7	<0.0001
**Liver**	96.4 ^b^	2.3	93.1 ^b^	1.9	128.9 ^a^	1.6	92.6 ^b^	7	110.7 ^ab^	7.5	<0.0001
**Total Se**	133.1 ns	3.8	138.6 ns	4.3	162.3 ***	6.9	125.1 ns	3.9	151.9 ***	5.7	0.0007

Data are presented as mean ± SEM. Different superscript letters within each row indicate statistically significant differences among dietary treatments (*p* < 0.05). Total selenium content was analyzed separately; asterisks indicate significant differences compared with the control (*** *p* < 0.001), while ns denotes non-significant differences.

**Table 3:** Total selenium retention and depletion rates in Japanese quails following SeNP supplementation and a 7-day withdrawal period.

	C	T1	T2	T3	T4
**Total** **Se retention %**	96%	91%	78%	88%	57%
**Total Se** **Depletion %**	4%	9%	22%	12%	43%

## Data Availability

The data supporting the findings of this study are contained within the article.

## Abbreviations

The following abbreviations are used in this manuscript:

SeNPs	Selenium nanoparticles
GPx	Glutathione peroxidase
SOD	Superoxide dismutase
TAC	Total antioxidant activity
RBF	Red blood fraction
Se	Selenium
AFS	Atomic Fluorescence Spectroscopy
